# Does place matter in the implementation of an evidence‐based program policy in an Australian place‐based initiative for children?

**DOI:** 10.1111/hsc.14010

**Published:** 2022-09-08

**Authors:** Fiona C. Burgemeister, Stacey Hokke, Sharinne B. Crawford, Naomi J. Hackworth, Jan M. Nicholson

**Affiliations:** ^1^ Judith Lumley Centre, School of Nursing & Midwifery La Trobe University Bundoora Victoria Australia; ^2^ Parenting Research Centre East Melbourne Victoria Australia; ^3^ Murdoch Children's Research Institute Parkville Victoria Australia; ^4^ Queensland University of Technology Brisbane Queensland Australia

**Keywords:** children, community, evidence‐based policy, family and child health, inequalities

## Abstract

Policy‐mandated requirements for use of evidence‐based programs (EBP) in place‐based initiatives are becoming more common. Little attention has been paid to the geographic aspects of uneven market development and urbanicity in implementing EBPs in large place‐based initiatives. The aim of this study was to explore geographic variation in knowledge, attitudes, and experiences of service providers who implemented an EBP policy in Australia's largest place‐based initiative for children, Communities for Children. A cross‐sectional online survey of Communities for Children service providers was conducted in 2018–2019, yielding 197 participants from all of Australia's eight states and territories. Relationships between two measures of ‘place’ (thick and thin market states; urbanicity: urban, regional and remote) and study‐designed measures of knowledge, attitudes, and implementation experiences were analyzed using adjusted logistic and multinomial regressions. Participants from thin market states (outside the Eastern Seaboard) were more resistant to the policy and experienced greater implementation challenges than those from thick market states (Eastern Seaboard). Regional participants reported greater knowledge about EBPs but experienced greater dissatisfaction and implementation challenges with the policy than both urban and remote participants. Our study found that place *does* matter when implementing EBPs in a place‐based initiative.

**What is known about this topic**

- Policy mandates can help accelerate the use of evidence‐based interventions; however, implementation requires careful consideration to ensure program effectiveness and sustainability.
- Uneven geographic development in Australia has created ‘thin’ markets, resulting in a scarcity of resources to support implementation in some areas.
- Little is known about how ‘place’ influences the implementation of evidence‐based programs (EBPs) in place‐based initiatives.

**What this paper adds**

- Place is a key factor in service providers' knowledge, attitudes, and experiences of implementing EBPs in Australia's largest place‐based initiative for children. Services located in geographically ‘thin’ market states and non‐urban areas face greater barriers to policy implementation than those in ‘thick’ market states and urban locations.
- The policy environment must recognize and respond to the unique service delivery context in ‘thin’ markets brought about by uneven geographic development.

## 1 INTRODUCTION

Socio‐economic disadvantage is geographically concentrated (Hertzman & Keating, 1999), and children living in disadvantaged neighborhoods have comparatively poorer health and development outcomes than peers living in advantaged neighbourhoods (Edwards & Bromfield, 2010; Phillips & Shonkoff, 2000). Place‐based approaches target defined geographic areas to reduce inequalities in child, family, and community outcomes using programs and services to address local need (McLachlan et al., 2013). These place‐based approaches are typically founded on the core principle of community autonomy in program selection, although policy‐mandated requirements for the use of evidence‐based programs (EBPs) are often introduced (Compass Evaluation and Research, 2015; Goff et al., 2013; Robinson, 2017). Evidence as to what factors influence the successful implementation of EBPs within large‐scale place‐based initiatives is limited. This study explores whether ‘place’ is an implementation factor by examining geographical variations in the knowledge, attitudes and implementation experiences of service providers following the introduction of an EBP requirement in the Communities for Children Facilitating Partners Program (CfC), an Australian place‐based initiative for children.

Many publicly funded community interventions have never been evaluated (Bumbarger & Perkins, 2008). While there is growing evidence for which programs are effective in addressing child and family concerns in community settings and a proliferation of models and frameworks to guide implementation (Damschroder et al., 2009; Fixsen et al., 2005, 2013; Greenhalgh, 2018; Tabak et al., 2012), the gap between this knowledge base and implementation of EBPs into community practice remains large. Factors driving the research‐to‐service gap include: poor communication about what works; staff resistance to change; practical obstacles that diminish program impact (Durlak & DuPre, 2008); perceived time and financial burdens of introducing EBPs (Ramanadhan et al., 2012); and inadequate investment in implementation (Fixsen et al., 2013). For community‐wide initiatives, additional challenges include poor transference to different settings, lack of community acceptance, and negative service provider attitudes (Ghate, 2018; Weiss et al., 2008). Drivers of successful implementation span a range of program, workplace, process and interaction factors (Bumbarger & Perkins, 2008; Hodge & Turner, 2016) including selecting and recruiting the right staff, access to training and supervision, management support, ongoing technical support, system‐level partnerships, and evaluation (Metz et al., 2007). At the community level, all drivers are not always available, for example, adequately qualified staff and may be compensated for by other drivers such as strong leadership and organisational support (Fixsen et al., 2016).

Governments are increasingly using policy mandates or funding mechanisms to increase EBP use in community settings (US Department of Health and Human Services, 2018), yet there is uneven geographical distribution of the necessary staff and support structures needed for implementation success. The *political economy approach* to health geography highlights the influence of macro‐level political, economic, and structural factors in creating geographical inequalities (Bambra et al., 2019). For large countries with unequally distributed populations, urbanicity is a key macro‐level factor (Girth et al., 2012; Warner, 2006). Ample evidence has demonstrated the dearth of health services and poorer health outcomes experienced by regional and remote communities than their urban counterparts (Roufeil & Battye, 2008; Statz & Evers, 2020). Macro‐level economic policy can also lead to geographic variation at a broader (i.e. state or regional) level. For example, Bambra et al. (2019) describe how deliberate policy intervention led to a rapid closing of the health gap between East and West Germany following reunification, while in England, policy decisions have resulted in persistent health inequities between the North and South.

In Australia, state‐based differences in resources and governance interact with the market‐based approach to public service delivery used by governments to promote choice and competition (Carey et al., 2017). Macro‐level influences have resulted in geographic variations in the ‘market thickness’ of public services (Girth et al., 2012). ‘Market thickness’ refers to the level of competition in the market and can apply to the workforce, services, and products. ‘Thin markets’ are sometimes referred to as ‘noncompetitive markets’ (Girth et al., 2012; Warner, 2006). When applied to public services, thick markets have a large and diverse array of services and staff from which to choose, while thin markets have more difficulty accessing the services, staff, and technical support necessary to build capacity. In Australia, thick markets are more likely located along the Eastern Seaboard comprising the most heavily populated states and largest cities (New South Wales, Victoria, Queensland) and the centre of the federal government (Australian Capital Territory). Thin markets are more likely to occur in the remaining states and territories (Northern Territory, South Australia, Western Australia, Tasmania), which have a larger combined land mass, smaller populations, and greater distance from the federal government centre (O'Neill & McGuirk, 2002).

**FIGURE 1:**
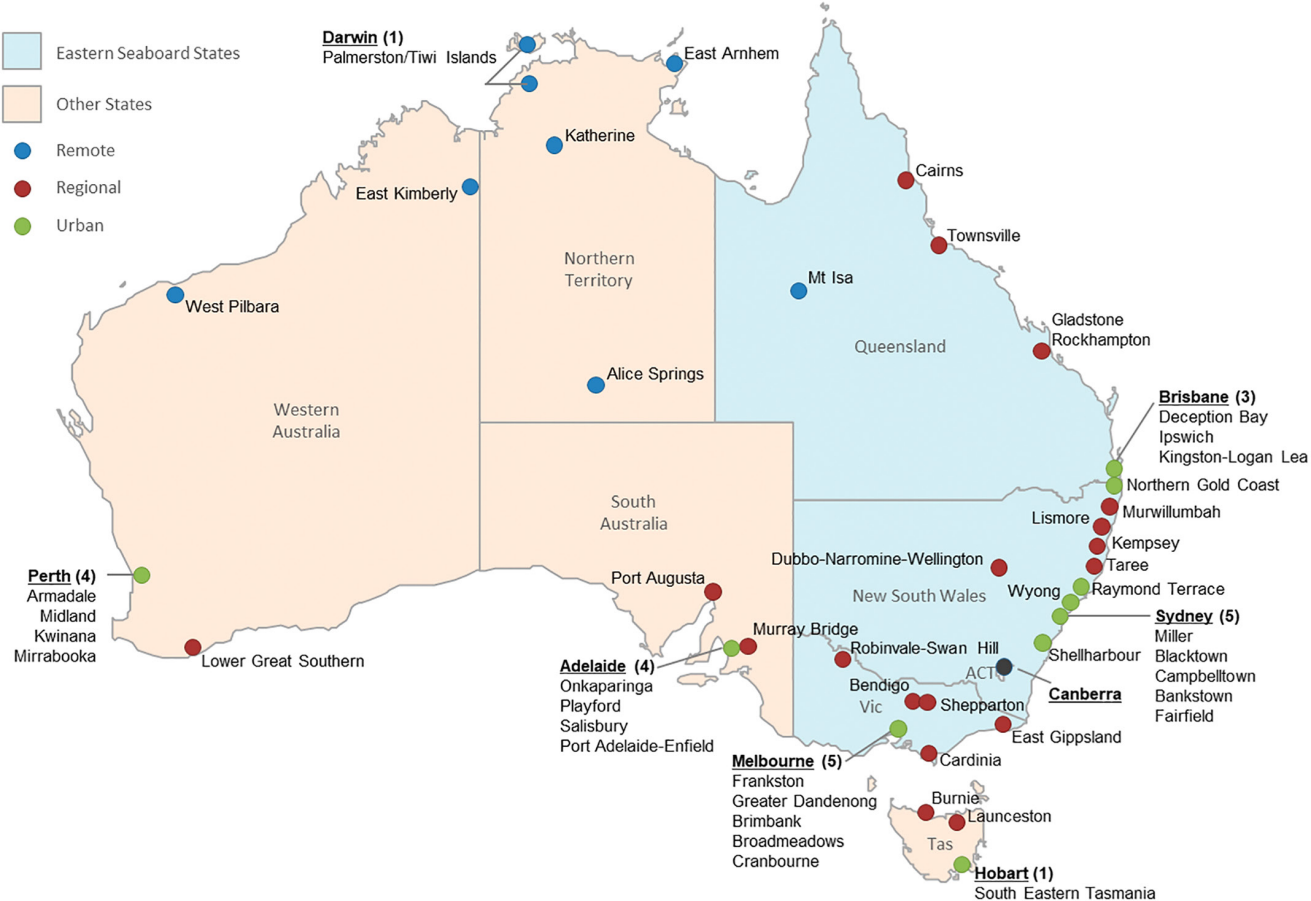
Map of communities for children sites

Established by the Australian government in 2004, CfC is a place‐based initiative to support children (birth to 12 years) and families in 52 disadvantaged communities (‘sites’) across Australia (Figure 1). On average, CfC sites are characterised by higher rates of unemployment and lone parent households, lower education levels, and greater cultural diversity including high numbers of indigenous families (Katz et al., 2007). In 2015, a policy was introduced requiring 30% of direct service delivery funding be spent on approved EBPs, rising to 50% by 2017. Sites could meet this using pre‐approved programmes listed in the CfC Guidebook, or by collecting evaluation evidence for their home‐grown programmes to be classified as ‘Promising’, see Table 1 for criteria. Sites are responsible for selecting and funding programmes, including any associated staffing and training. Prior to the introduction of the policy, there were no programme stipulations, other than they met identified local needs. Programmes are varied and include parenting and family support, interventions with children to support their cognitive, behavioural and emotional development, school readiness support, and interventions with at‐risk or vulnerable families. Programmes are provided in community, school and home settings. The Australian Department of Social Services (DSS), located in Canberra, Australian Capital Territory, administers CfC, and the Australian Institute of Family Studies, located in Melbourne, Victoria, provides central education and practice support to sites.

**TABLE 1:** Criteria for programmes to be included as part of 50% evidence‐based quota in communities for children

Evidence‐based program criteria A programme needs to meet the following criteria to be classed as evidence‐based in CfC: The objectives of the programme are in line with the objectives of the CfC Facilitating Partner model. The programme is primarily targeted at children aged 0–12 years and their families. The following documented information about the programme is readily available: aims, objectives and a theoretical basis for the programme; a programme logic or similar; the target group for the programme is clearly articulated; and elements/activities of the programme and why they are important. The programme should include a training manual or documentation that allows for replication within Australia. Evaluation of the programme has been undertaken with the following characteristics: Impact: At least one high‐quality evaluation has been conducted that showed positive impacts on the desired outcomes of the program(s), and no negative effects were found. The programme must have been evaluated in a cultural setting that is similar to Australia. Design (one or more of): A randomised controlled trial or quasi‐experimental design that has a sample size of at least 20 participants in each of the intervention and control groups. High‐quality qualitative evaluation that includes at least 20 participants. The assessment of quality relies on availability of information about factors such as the selection/inclusion/recruitment processes, the nature and representativeness of the sample, the process for administering data collection tools, and the degree of independence from the programme developer/implementer. A high‐quality combination of the above (mixed methods).
b Promising program criteria Promising programmes must meet the following 5 criteria, representing minimum standards for a quality programme: A programme must have a documented theoretical and/or research background. A programme must have a clear program logic, which reflects good practice both in terms of logical pathways from activities to outcomes and in meeting the needs of the intended target group. The activities undertaken in the programme are documented, and activities generally match good practice in addressing the needs of the target group. One or more evaluations of the programme have been conducted (with a minimum total of 20 participants) that establishes the programme as having positive benefits for the target group, with at least pre‐AND post‐testing of participant outcomes (ideally with a validated outcomes measurement tool), and a report is available. Staff members that run the programme are sufficiently qualified and/or trained.

*Note*: Australian Government (2019).

Place‐based initiatives such as CfC are founded on an understanding of the health impacts of local place‐based variations in socioeconomic factors. Specifically, they focus on disadvantaged communities and seek to improve child health outcomes by providing programmes and services selected to meet identified community needs. When implementing such approaches, scant attention has been given to the potential effects of broader geographic differences, such as macro‐level variations by state or urbanicity. Additionally, few studies have directly examined the experiences of service providers implementing EBPs in place‐based initiatives via a public policy mandate and those that do are largely confined to checking programme fidelity (Goff et al., 2013). For example, in CfC, the Australian government has mandated that while communities can take a place‐based approach to program choice, 50% of funding must be spent on EBPs. Our recent qualitative study (Burgemeister et al., 2022) found that government personnel tasked with overseeing implementation of the EBP policy in CfC perceived implementation as more challenging outside Eastern Seaboard states and in regional and remote areas due to limited staff availability and high staff turnover and the concentration of training and support services in major cities on the Eastern Seaboard (Figure 1).

### 1.1 The current study

This study examines whether there are macro‐level ‘place’ differences in the knowledge, attitudes, and experiences of service providers implementing an EBP policy in CfC. The current study was conducted with two types of CfC personnel: *Facilitating Partner* employees responsible for *coordinating* site‐wide CfC activities including the *selection* of programmes; and *Community Partner* employees responsible for *delivering* CfC programmes to families. The study aimed to identify macro‐level geographic variations in knowledge, attitudes, and implementation experiences of the EBP policy, using two lenses: place defined by Eastern Seaboard vs non‐Eastern Seaboard state grouping (i.e. thick vs thin market states); and place defined by urbanicity (urban vs regional vs remote).

## 2 METHODS

### 2.1 Study design

A cross‐sectional online survey was conducted between November 2018 and September 2019. Active recruitment occurred from November to December 2018 and July to September 2019. Review of participant characteristics following the first recruitment period revealed low participation from Community Partners. Communications for the second recruitment period were tailored to Community Partner participants. The survey was administered via Qualtrics (www.qualtrics.com) and was approved by the La Trobe University Human Research Ethics Committee (HEC18198) and DSS.

Participants were eligible to participate if they worked in a CfC Facilitating Partner or Community Partner organisation. Given their occupation, it was assumed participants were over the age of 18 with sufficient English to complete the survey. Participation was anonymous, and potentially identifiable data were coded before analysis to avoid inadvertent identification.

### 2.2 Recruitment

A cascade approach to recruitment was used, where DSS sent email invitations, including the survey link, to the 52 Facilitating Partners for further distribution to their staff and to their Community Partners. Consent was obtained electronically within the survey.

### 2.3 Measures

The survey comprised study‐developed measures to assess service providers’ knowledge, attitudes, and implementation experiences of the CfC EBP policy (Table 2). These measures were directly informed by our earlier qualitative study and refined through consultation with key DSS personnel. Measures were knowledge about EBPs; understanding of policy rationale; attitude towards the policy and the 50% target; programme fidelity confidence; programme fidelity capacity building support; adequacy of Guidebook programme range; limitations in Guidebook programme fit; adverse impact on programme offerings; and staff and training challenges. To obtain additional insights, three open‐ended questions asked: “Is there anything else you would like to tell us?”, “…about the 50% requirement?”; and “…about your experiences of evaluation?” The survey was piloted with three CfC sites with minor modifications made to improve clarity and reduce length.

**TABLE 2:** Measures of knowledge, attitudes, and implementation experiences

Measure	Items and response options	Internal reliability	Categories used in analysis (score range); response distribution
Knowledge			
Knowledge about evidence‐based programs	2 items: “I understand what is meant by the term ‘evidence‐based’ in Communities for Children (CfC)”; “I am aware of which programs in my CfC meet the evidence‐based program requirement”. Rated: 1 “Not at all”, 2 “Somewhat/partially”, 3 “Yes ‐ good knowledge”.	*r* = 0.59	Summed item scores: Good (6); 76.4% Other (1–5); 23.6%
Understanding of policy rationale	Single item: “I understand the rationale for this policy”. Rated: 1 “Strongly disagree” to 5 “Strongly agree”.	n/a	Single item scores: poor/fair (1–3); 10.3% good (4, 5); 89.7%
Attitudes			
Attitude towards the policy	5 items: E.g. “I support this policy”; “This policy has been a change for the better”. Rated: 1 “Strongly disagree” to 5 “Strongly agree”.	*α* = 0.91	Mean item scores: Resistant (≤2.5); 9.8% Ambivalent (2.5 to < 4); 58.2% Positive (≥4); 32.0%
Attitude towards the 50% target	Single item: “A target of 50% evidence‐based programs is too high”. Rated: 1 “Strongly disagree” to 5 “Strongly agree”.	n/a	Positive (1, 2); 28.4% Ambivalent (3); 39.6% Resistant (4, 5); 32.0%
Implementation experiences			
Program fidelity confidence	3 items: e.g. “I know how programs can be adapted and implemented with fidelity”. Rated: 1 “Not at all” to 5 “Very great extent”	*α* = 0.78	Mean item scores: Not at all to moderate (<4); 46.1% Great to very great (≥4); 53.9%
Program fidelity capacity building support	Single item: “I would like more information and support about program adaptation and implementation”. Rated: 1 “Not at all” to 5 “Very great extent”	n/a	Single item scores: Not at all to moderate (1–3); 62.2% Great to very great (4, 5); 37.8%
Adequacy of Guidebook program range	3 items: e.g. “We have had no trouble choosing programs from the Guidebook that meet our community's needs”; “The Guidebook does not have enough suitable programs for our families” Rated: 1 “Strongly disagree” to 5 “Strongly agree”. Two items reverse coded so that higher score indicates greater adequacy.	*α* = 0.84	Mean item scores: Range is not adequate (≤3); 81.2% Range is adequate (>3); 18.8%
Limitations in Guidebook program fit	Single item: “Meeting the 50% evidence‐based program requirement sometimes means selecting programs from the Guidebook that are not an ideal fit for our community's needs” Rated: 1 “Strongly disagree” to 5 “Strongly agree”	n/a	Single item scores: Strongly disagree to neither agree or disagree (1–3); 38.9% Agree or strongly agree (4, 5); 61.1%
Adverse impact on program offerings	Three items about the impact of the policy on the range of programmes on offer to communities: e.g., “This policy has meant we have ceased providing programs that worked for our families”. Rated: 1 “Strongly disagree” to 5 “Strongly agree”.	n/a^a^	Frequency count of items rated agree or strongly agree: Little impact (count = 0–1); 61.7% Adverse impact (count = 2–3); 38.3%
Staff and training challenges	Five items about staff recruitment, turnover, and cost/availability of training with the item stem “To what extent has your site/organisation experienced the following challenges to implementing evidence‐based programs?” E.g. “Finding suitable staff with the required skills to deliver evidence‐based programs”; “Limited availability of training” Rated: 1 “Not at all” to 5 “Very great extent”	n/a^a^	Frequency count of items rated great or very great: Low challenges (count = 0–1); 28.7% High challenges (count = 2–3); 71.3%

^a^ Internal consistency not applicable for checklists.

Two measures of place were used. *State* was classified as Eastern Seaboard (New South Wales, Australian Capital Territory, Victoria, Queensland) or Other (South Australia, Western Australia, Northern Territory, Tasmania). *Urbanicity* is measured by the ‘relative remoteness’ of the site location using the Accessibility and Remoteness Index of Australia (Australian Bureau of Statistics, 2018), derived by measuring the road distance to the nearest urban centre in five population ranges (major city, inner regional, outer regional, remote, very remote). The two regional and two remote categories were combined to create three population ranges for sites: urban (located in suburbs in or adjacent to major cities), regional (located in medium to large country towns and nearby communities), or remote (dispersed population located a vast distance from other cities or towns) (Figure 1). *Organisational* characteristics were organisation type (Facilitating Partner; Community Partner); organisation scale (national [large Australia‐wide, multi‐site organisation with broad remit]; state/small national [single state, multi‐site organisation with broad remit or Australia‐wide organisation with single program focus]; local [small, local or regionally focussed organisation]); and level of evaluation support (low [no support or single support from contractors as required]; high [multiple types of support or single ongoing support from inhouse research and evaluation, university or external organisation]). *Individual* characteristics were: gender; age; years working for CfC (≤3, ≥4); hours per week (part‐time [<28 h]; full‐time [≥28 h]); and role (manager/coordinator; direct service provider; other).

### 2.4 Participants

Of 245 people who consented and commenced the survey, 48 (19.6%) dropped out prior to questions about the EBP policy and were excluded from analysis. The final sample comprised 197 participants: 70 from Facilitating Partner organisations and 127 from Community Partner organisations.

### 2.5 Data preparation and analysis

Statistical analyses were performed using Stata 16 SE. Knowledge, and attitudes and implementation experiences were assessed using multi‐item and single‐item measures (Table 2). Two multi‐item measures were derived as frequency counts: adverse impact on programme offerings (three items) and staff and training challenges (five items). For the remainder, internal reliability was examined using Cronbach's alpha and inter‐item correlations. Imputation (into the neutral‐ or mean‐level) was undertaken to account for item‐level missing data (if <30% of items missing per measure). Tests for normality showed most measures were skewed with values close to a natural limit. Continuous and ordinal Likert scales were converted to two‐ or three‐group categories and cut‐points determined based on response distributions and to allow for meaningful interpretation (Table 2). All dependent variables used in analysis are categorical.

Summary descriptive statistics were used to describe the place‐based, organisational, and individual characteristics of the sample, stratified by organisation type (Facilitating Partner and Community Partner). Relationships between the two place‐based factors (state and urbanicity) and all measures of knowledge, attitudes, and implementation experiences were initially analysed using chi‐square test. For multi‐item measures, logistic regressions were conducted to further evaluate differences by place. Binary logistic regressions were performed separately for dichotomous dependent variables (knowledge about EBPs, fidelity confidence, guidebook programme range, adverse impact on program offerings, staff, and training challenges), and a multinomial regression was performed for one variable (attitudes towards policy change: positive, negative and ambivalent), with ‘ambivalent’ as the comparison group. For each regression, place‐based factors (state, urbanicity) were entered into the model (Model 1) and then adjusted for relevant individual and organisation characteristics (role, hours per week working at CfC, years working at CfC, organisation type and education level; Model 2). Regression data are presented as adjusted odds ratios (aOR) and adjusted relative risk ratios (aRRR) with 95% confidence intervals (CI). All models were repeated with inclusion of an interaction term between the two place‐based factors. No significant interactions were found and results are omitted for brevity.

Qualitative responses to open‐ended questions were provided by 78 respondents (61%) and exported to NVivo for directed content analysis, reported elsewhere (Burgemeister, 2022). This is a deductive method that helps extend knowledge or understanding of existing theory or prior research (Hsieh & Shannon, 2005). The lead author reviewed all responses and applied the codes and categories generated by our previous qualitative study of government‐level CfC staff to the data (Burgemeister et al., 2022). Text that could not be coded with the predetermined coding scheme was given a new code, and some codes and categories were re‐worded to better‐reflect survey responses. Coding was confirmed for 10% of responses by the third author, with a high degree of agreement (~80%). Content illustrating the constructs examined in this study were extracted, and verbatim quotes are presented throughout the result section to elucidate participants' views and experiences.

## 3 RESULTS

### 3.1 Sample characteristics

Characteristics of participants from Facilitating Partner organisations (*n* = 70) and Community Partner organisations (*n* = 127) are presented in Table 3. Most were female, born in Australia and had a Bachelor degree or higher. Half (53%) had been working in CfC for 3 years or less and around 20% were direct service providers. Participants from Facilitating Partner organisations were more likely than those from Community Partner organisations to work full‐time, to have worked in CfC for longer, and to work in a large, national organisation rather than in a state/small national or local organisation.

**TABLE 3:** Sample characteristics by organisation type

	All participants (*n* = 197), *n* (%)^a^	Facilitating partner organisation (*n* = 70), *n* (%)	Community partner organisation (*n* = 127), *n* (%)
Place characteristics			
State			
SA	25 (12.7)	11 (15.7)	14 (11.0)
NSW/ACT	42 (21.3)	13 (18.6)	29 (22.8)
VIC	54 (27.4)	16 (22.9)	38 (29.9)
WA	26 (13.2)	13 (18.6)	13 (10.2)
TAS	7 (3.6)	4 (5.7)	3 (2.4)
QLD	26 (13.2)	8 (11.4)	18 (14.2)
NT	15 (7.6)	4 (5.7)	11 (8.7)
State grouping			
Eastern Seaboard	122 (61.9)	37 (52.9)	85 (66.9)
Other	73 (37.1)	32 (45.7)	41 (32.3)
Urbanicity^b^			
Urban	93 (47.2)	34 (48.6)	59 (46.5)
Regional	70 (35.5)	28 (40.0)	42 (33.1)
Remote	32 (16.2)	7 (10.0)	25 (19.7)
Site and organisational characteristics			
Organisation scale			
National	102 (51.8)	58 (82.9)	44 (34.7)
State/small national	27 (13.7)	7 (10.0)	20 (15.8)
Local	68 (34.5)	5 (7.1)	63 (49.6)
No. of people in org working on CfC^c^ (FTE)			
<2	71 (36.0)	14 (20.0)	57 (44.9)
2–3	80 (40.6)	43 (61.4)	37 (29.1)
4–5	14 (7.1)	8 (11.4)	6 (4.7)
>5	19 (9.6)	4 (5.7)	15 (11.8)
Research and evaluation support^d^			
In‐house	90 (45.7)	45 (64.3)	45 (35.4)
University	84 (42.6)	36 (51.4)	48 (37.8)
External organisation	80 (40.6)	31 (44.3)	49 (38.6)
Contracts as required	124 (62.9)	55 (78.6)	69 (54.3)
Other^e^	39 (19.8)	13 (18.6)	26 (20.5)
None	31 (15.7)	7 (10.0)	24 (18.9)
Individual characteristics			
Gender^f^			
Male	28 (14.2)	9 (12.9)	19 (15.0)
Female	159 (80.7)	58 (82.9)	101 (79.5)
Age in years			
18–34	29 (14.7)	7 (10.0)	22 (17.3)
35–44	54 (27.4)	20 (28.6)	34 (26.8)
45–54	66 (33.5)	26 (37.1)	40 (31.5)
≥55	38 (19.3)	12 (17.1)	26 (20.5)
Country of birth			
Australia/New Zealand	152 (77.2)	55 (78.6)	97 (76.4)
Other	28 (14.2)	9 (12.9)	19 (15.0)
Education level			
Year 12, certificate, diploma	56 (28.4)	16 (22.9)	40 (31.5)
Bachelor degree, grad diploma/certificate	87 (44.2)	32 (45.7)	55 (43.3)
Post‐graduate degree	47 (23.9)	20 (28.6)	27 (21.3)
Professional background			
Education	33 (16.8)	10 (14.3)	23 (18.1)
Community Services	87 (44.2)	38 (54.3)	49 (38.6)
Social work, allied health, psychology	38 (19.3)	8 (11.4)	30 (23.6)
Other (incl multiple)	31 (15.7)	11 (15.7)	20 (15.8)
Years working at CfC			
≤3	104 (52.8)	34 (48.6)	70 (55.1)
4–6	55 (27.9)	16 (22.9)	39 (30.7)
≥7	38 (19.3)	20 (28.6)	18 (14.2)
Hours per week working at CfC			
Part‐time (<28 h)	100 (50.8)	19 (27.1)	81 (63.8)
Full‐time (≥28 h)	97 (49.2)	51 (72.9)	46 (36.2)
Role in CfC			
Manager, coordinator, supervisor	125 (63.5)	50 (71.4)	75 (59.1)
Direct service provider	41 (20.8)	0 (0.0)	41 (32.3)
Other^g^	31 (15.7)	20 (28.6)	11 (8.7)

^a^ Prefer not to say/missing categories omitted; some percentages do not total 100%.

^b^ Urbanicity is based on whether participants were working in remote/regional sites. Where participants worked across more than one site with differing densities, remote was prioritised first then regional then urban.

^c^ CfC, Communities for Children.

^d^ Participants could select more than one category.

^e^ Includes evaluation data submitted to the Australian Department of Social Services (DSS), pro bono evaluation support, assistance from the Australian Institute of Family Studies (AIFS), those who did research and evaluation in‐house without a dedicated research unit, and participants who were unsure.

^f^ Non‐binary, intersex, unspecified omitted.

^g^ Includes admin, data entry, research and evaluation, project/program support, community development, and contract support.

Participants were employees from 50 of the 52 CfC sites. Half (52%) were from regional and remote sites, and 62% were from Eastern Seaboard states. No centralised, consolidated record of CfC personnel is available for determining sample representativeness. However, participation by urbanicity (47% urban, 36% regional, 16% remote) was proportionally similar to the distribution of sites across Australia (52% urban, 35% regional, 13% remote). Participation by state was proportionally similar to the distribution of sites across Australian states and territories (ACIL Allen Consulting, 2016), with Queensland somewhat underrepresented (13% of participants compared to 17% of sites) and Victoria overrepresented (27% of participants compared to 19% of sites).

### 3.2 Knowledge, attitudes and experiences: Multi‐item measures

In this section, we describe the geographic variations in knowledge, attitudes, and experiences for all multi‐item measures, with proportions presented in Table 4 and logistic and multinomial regressions in Table 5. Findings for single‐item measures of knowledge, attitudes, and experiences are presented in the subsequent section.

**TABLE 4:** Multi‐item measures: Proportions for knowledge, attitudes, and experiences of the evidence‐based policy

	Total, *n* (%)	Urbanicity				State		*p* (χ^2^)
		Urban, *n* (%)	Regional, *n* (%)	Remote, *n* (%)	*p* (χ^2^)	Eastern seaboard, *n* (%)	Other, *n* (%)	
Knowledge of evidence‐based policy^1^								
Good	149 (76.4)	67 (72.0)	61 (87.1)	21 (65.6)	**0.02**	95 (77.9)	54 (74.0)	0.54
Attitude towards policy^2^								
Resistant	19 (9.8)	7 (7.5)	11 (15.7)	1 (3.1)	0.08	8 (6.6)	52 (42.6)	**<0.0001**
Positive	62 (32.0)	34 (37.6)	21 (30.0)	7 (21.9)		11 (15.1)	11 (15.1)	
Program fidelity confidence^3^								
Great, very great	104 (53.9)	49 (52.7)	42 (61.8)	12 (40.0)	0.13	73 (61.3)	30 (41.7)	**0.008**
Guidebook program range^4^								
Adequate	32 (18.8)	10 (11.9)	12 (20.3)	10 (40.0)	**0.007**	23 (21.5)	9 (14.8)	0.29
Adverse impact on program offerings^5^								
Adverse	69 (38.3)	36 (42.4)	28 (41.8)	5 (18.5)	0.07	39 (34.8)	30 (44.8)	0.19
Staff and training challenges^5^								
High	129 (71.3)	56 (65.1)	54 (81.8)	19 (65.5)	0.06	79 (71.8)	50 (70.4)	0.84

*Note*: Comparison group(s): ^1^poor/fair; ^2^ambivalent; ^3^not at all to moderate; ^4^inadequate; ^5^Low.

Bold text indicates *p* < .05.

**TABLE 5:** Logistic and multinomial regressions for knowledge, attitudes, and experiences of the evidence‐based policy

	Urbanicity			State	
	Urban	Regional	Remote	Eastern seaboard	Other
“Good” knowledge of policy					
OR	ref	2.67	0.78	ref	0.89
Adjusted OR^a^ (95% CI)	ref	3.00 (1.14, 7.89)	2.00 (0.60, 6.5)	ref	0.63 (0.26, 1.57)
*p*_aOR_		**0.03**	0.26		0.33
“Resistant” attitude towards policy					
RRR	ref	1.95	0.21	ref	2.23
Adjusted RRR^a^ (95% CI)	ref	2.72 (0.83, 8.87)	0.36 (0.03, 3.36)	ref	3.59 (1.01, 12.84)
*p*_aRRR_		0.097	0.39		**0.049**
“Positive” attitude towards policy					
RRR	ref	0.88	0.84	ref	0.27
Adjusted RRR^a^ (95% CI)	ref	0.86 (0.40, 1.86)	0.88 (0.26, 2.96)	ref	0.27 (0.11, 0.64)
*p*_aRRR_		0.71	0.83		**0.003**
“Good” program fidelity confidence					
OR	ref	1.61	0.92	ref	0.89
Adjusted OR^a^ (95% CI)	ref	1.77 (0.88, 3.57)	1.11 (0.40, 3.08)	ref	0.50 (0.25, 1.02)
*p*_aOR_		0.11	0.85		0.055
“Adequate” Guidebook program range					
OR	ref	2.29	13.05	ref	0.22
Adjusted OR^a^ (95% CI)	ref	2.55 (0.88, 7.37)	15.49 (3.03, 79.27)	ref	0.19 (0.05, 0.73)
*p*_aOR_		0.08	**0.001**		**0.02**
Adverse impact on program offerings					
OR	ref	0.88	0.18	ref	2.36
Adjusted OR^a^ (95% CI)	ref	1.09 (0.52, 2.3)	0.24 (0.07, 0.85)	ref	2.24 (1.01, 4.98)
*p*_aOR_		0.80	**0.03**		**0.048**
“High” staffing and training challenges					
OR	ref	2.42	1.05	ref	0.95
Adjusted OR^a^ (95% CI)	ref	2.93 (1.23, 6.96)	0.88 (0.29, 2.70)	ref	0.90 (0.39, 2.10)
*p*_aOR_		**0.015**	0.82		0.82

^a^ Adjusted for: role, hours at Communities for Children, years at Communities for Children, organisation type, education level.

Bold text indicates *p* < .05.

‘Good’ knowledge about EBPs was reported by three‐quarters of participants (76%) (Table 4), with statistically significant differences by urbanicity but not by state. After adjustment for other factors (Table 5), the odds of reporting good knowledge were three times higher for regional participants than urban participants (aOR: 3.00; 95% CI: 1.14–7.89; *p* = 0.03). Resistance towards the evidence‐based policy was expressed by a small proportion of participants with differences by state but not by urbanicity. In the adjusted multinomial regression (Table 5), relative to having ‘ambivalent’ attitudes (comparison group), participants outside the Eastern Seaboard states were more likely to have negative (‘resistant’) attitudes (aRRR: 3.59; 95% CI: 1.01–12.84; *p* = 0.049) and less likely to have ‘positive’ attitudes (aRRR: 0.27; 95% CI: 0.11–0.64; *p* = 0.003) than participants from other states.

Attitudes towards the EBP policy as expressed in the open‐ended questions revealed a mix of positive, ambivalent, and negative views. Positive comments highlighted the opportunities for learning, collaboration, and benefits for families. Ambivalent or negative views described frustration with the policy implementation and concerns about meeting local needs:

I have enjoyed the process. It ensures we remain relevant and families are receiving the most up to date research. (Eastern Seaboard State, Urban Community Partner)

It has been incredibly frustrating and at times a complete waste of time and energy working with various strangers sent over from the east coast (Other State, Remote Community Partner)

It has limited [our] time to develop new innovative ideas that are reflective of our unique community. EBP is a little bit like One Size Fits All. (Eastern Seaboard State, Regional Facilitating Partner)

Confidence about programme fidelity and adaptation was reported by 54% of participants (Table 4). Differences by place did not remain in the adjusted model (Table 5). Participants commented in the open‐ended questions that adaptations were necessary for programmes to succeed, but they lacked specific evidence and government support to do this. Ongoing support was reported to be valuable but costly:

The most successful implementations with strong fidelity have been where there is capacity to "check in with experts" post training for questions of adaptation and its impact on fidelity during implementation…This is a more expensive model…but the quality and results are more reliable. (Eastern Seaboard State, Urban Facilitating Partner)

The ‘adequacy’ of the Guidebook program range was reported by only 19% of participants (Table 4). In the adjusted logistic regression model (Table 5), both urbanicity and state predicted adequacy: the odds of reporting adequate Guidebook program range were higher for remote than urban participants (aOR: 15.49; 95% CI: 3.03–79.27; *p* = 0.001), while the odds were lower for participants from Other states than Eastern Seaboard states (aOR: 0.19; 95% CI: 0.05–0.73; *p* = 0.02).

Many participants commented on perceived limitations of the Guidebook programme range. Current programmes were heavily focussed on parenting, and viewed as unsuitable for diverse and complex families:

Many of the EBPs are centred around parenting programmes – it has been difficult to get parents to commit to a number of weeks … especially when travel for them is involved.” (Other State, Regional Facilitating Partner)

…[DSS] constantly ask for the same old programmes that are manual and paperwork based for families who don't read or write (Eastern Seaboard State, Regional Community Partner)

Some of the programmes in the Guidebook can 'do harm' to families as they are not suitable for certain populations – particularly rural, remote, Aboriginal, CALD [cultural and linguistically diverse], and complex trauma. (Other State, Regional Facilitating Partner)

Adverse impacts of the policy on programme offerings were reported by 38% of participants (Table 4). Both state and urbanicity were predictors in the adjusted logistic regression model (Table 5). The odds of the policy having an adverse impact on programme offerings was lower for remote than urban participants (aOR: 0.18; 95% CI: 0.07–0.85; *p* = 0.03), while the odds were higher for participants outside the Eastern Seaboard than Eastern Seaboard participants (aOR: 2.36; 95% CI 1.01–4.98; *p* = 0.048).

A high level of staff and training challenges (defined as two or more challenges) associated with implementing the policy was reported by 71% of participants (Table 4). In the adjusted logistic regression analysis (Table 5), the odds of reporting a high number of challenges were 2.42 times greater for regional than urban participants (95% CI: 1.23–6.96; *p* = 0.02).

Many respondents commented about staffing and training issues such as staff turnover, high training costs, the background skill set of staff which did not prepare them for this type of work, and difficulty accessing external support to supplement internal skill deficits:

Unforeseen staff turnover and the high cost of retraining new staff in EBPs has been difficult for CfC and our funded community partner organisations to manage. (Eastern Seaboard State, Urban Facilitating Partner)

Some programmes in the Guidebook limit Community Partners due to the qualifications/training required by an individual to be able to implement the program, eg, music therapist, university degree, and psychologist. Not for profit organisations in rural communities can struggle to afford employing someone with these qualifications (Other State, Regional Community Partner)

CFC FPs are predominately Community Development backgrounds and not evaluators but have had to adapt very quickly to this huge shift. We received limited training and support around this. (Other State, Regional Facilitating Partner)

### 3.3 Knowledge, attitudes, and experiences: Single‐item measures

Proportions for single‐item measures of knowledge, attitudes, and experiences are presented in Table 6. ‘Good’ understanding of the rationale for the policy was reported by most participants (90%), with no difference by place. While ‘resistant’, ‘ambivalent’, and ‘positive’ attitudes towards the 50% target for EBPs were each reported by around a third of all participants, remote sites had a significantly smaller proportion of ‘resistant’ participants (10%) than their urban (32%) and regional (41%) counterparts (*p* < 0.0001). Overall, 38% of participants wanted more information and support about program adaptation and implementation, with no difference by place. Over 60% of participants agreed or strongly agreed that there were limitations with the fit of some Guidebook programmes, again with no difference by place.

**TABLE 6:** Single item measures: proportions of knowledge, attitudes and implementation experiences

	Total, *n* (%)	Urbanicity				State		
		Urban, *n* (%)	Regional, *n* (%)	Remote, *n* (%)	*p* (*χ* ^2^)	Eastern seaboard, *n* (%)	Other, *n* (%)	*p* (*χ* ^2^)
Understanding of policy rationale^1^								
Good	177 (89.9)	85 (91.4)	61 (87.1)	29 (90.6)	.66	39 (86.7)	138 (90.8)	.42
Attitude towards 50% target^2^								
Resistant	62 (31.8)	30 (32.3)	29 (41.4)	3 (9.4)	**.002**	38 (31.2)	24 (32.9)	.12
Positive	56 (28.7)	28 (30.1)	21 (30.0)	7 (21.9)		41 (33.6)	15 (20.6)	
Program fidelity capacity building support^3^								
Great/very great	73 (37.8)	39 (41.9)	22 (32.4)	11 (36.7)	.46	42 (35.3)	30 (41.7)	.38
Limitations in Guidebook program fit^4^								
Agree/strongly agree	99 (61.1)	46 (57.5)	41 (70.7)	11 (47.8)	.11	57 (55.3)	41 (70.7)	.055

*Note*: Comparison group(s):^.1^Poor/fair; ^2^Ambivalent; ^3^Not at all to moderate; ^4^Strongly disagree/disagree/neither agree or disagree.

Bold text indicates *p* < .05.

All open‐ended comments about the 50% target expressed concern that it adversely impacted service provision or that an increase beyond the current target was on the policy horizon. Some participants voiced a preference for an “evidence‐informed” rather than “evidence‐based” approach to service delivery to allow greater flexibility in the types of programs that could be delivered:

50% impacts way too much on our service delivery…Evidence informed practice is where we need to focus. (Other State, Regional Facilitating Partner)

…[50%] is a good benchmark and I would not like to see it go any higher to leave room for innovation and place‐based programme development. (Eastern Seaboard State, Regional Facilitating Partner)

… if the [50%] were to increase we would need to change our partners solely based on this requirement as opposed to [their] great work or connections and relationships. (Eastern Seaboard State, Urban Facilitating Partner)

## 4 DISCUSSION

This novel study examined the impact of two aspects of place, ‘state’ and ‘urbanicity’, on the knowledge, attitudes, and experiences of service providers implementing EBPs for children, parents, and families in a large, complex place‐based initiative. Overall, we found evidence of both state and urbanicity differences in service providers' knowledge, attitudes, and experiences of implementing EBPs over and above the influence of personal or employment characteristics of individual respondents and their organisations. Supported by qualitative data, our study illustrates how services were adversely affected by their location in geographically thin markets.

In terms of state, personnel outside the Eastern Seaboard were more likely to be resistant to the EBP policy and report adverse program impacts than Eastern Seaboard participants and were less likely to consider the Guidebook programme range as adequate. These results align with the findings from our qualitative study where CfC government level personnel perceived that those working outside the Eastern Seaboard experienced greater challenges with accepting and implementing the policy. This struggle can, in part, be explained by the political economy whereby resources have become concentrated in ‘advantageous’ locations in Australia, leading to ‘thick’ and ‘thin’ market states (Bambra et al., 2019; O'Neill & McGuirk, 2002). The resistance reported in our study may speak to broader attitudes and experiences from service providers due to the centralisation of federal government services in Eastern Seaboard cities and the limited evaluation and program implementation support available locally. Previous research has shown that when resources are centralised, it reduces opportunities for all to participate in training and education activities (Schmidt et al., 2020).

In terms of urbanicity, participants in regional sites were more likely than urban participants to report good knowledge of EBP but were also more likely to report staffing and training challenges, and a greater proportion were resistant to the 50% EBP target. In contrast, participants from remote sites were less likely to report adverse programme impacts and more likely to report the programme range was adequate than those in urban locations, and a smaller proportion were resistant to the policy target. This is somewhat surprising as we had expected remote areas to report the greatest implementation challenges due to difficulties attracting and retaining a skilled workforce and accessing training and implementation support. It is possible that remote areas are used to working with limited resources, are more familiar with implementing programs in challenging environments, and are therefore more resourceful and adaptable. For example Fixsen et al. (2016) suggest that some organisations are able to use certain implementation drivers to compensate for deficiencies in other drivers. Thus, it may be that remote sites employed other drivers of implementation such as strong, adaptable leadership and an enthusiasm for using evidence in practice, where other drivers such as training and coaching and sharing of knowledge were less prevalent. Further exploration of these hypotheses is required.

Our findings highlight the importance of recognising and responding to broad geographical differences within a complex national initiative that is administered centrally but delivered locally. The first step is to address the knowledge and skill deficit outside the Eastern Seaboard and in non‐urban areas due to the limited availability of qualified staff and high turnover. Previous studies have found community‐based personnel in regional and remote areas are less qualified to use evidence‐based approaches (Patelarou et al., 2013). This suggests a need for regular and repeated provision of EBP education delivered in formats that all can access irrespective of location. There is also the need to find meaningful and sustained ways for producers (i.e. academics or researchers) of EBPs to interact and collaborate with service providers evenly across the initiative (Rycroft‐Malone et al., 2011).

Three elements critical to program sustainability, especially in disadvantaged communities are ‘good fit’ for the target community, available workforce, and ability for programs to be adapted to suit local context (Hodge & Turner, 2016). Previous studies have shown that adherence to key features of effective programmes (such as program length and structure) can be poor when EBPs are transferred to real‐world settings (Bumbarger & Perkins, 2008). This is when taking into account the local cultural, industrial, and landscape place differences becomes important. Overall, CfC areas are more disadvantaged than the rest of Australia, but each site has unique features that may include high Aboriginal and/or immigrant populations, high rates of family violence, transient populations centred around particular industries, and seasonal weather patterns that make service delivery challenging. Thus, both the macro perspectives highlighted in our findings and local perspectives are required when determining resource and support needs. We note that the uneven geographic distribution of available training and technical support for program adaption commonly observed in thin markets presents challenges. Our study found that participants perceived a limited range of Guidebook programmes and sometimes had to choose programmes that were not an ideal fit for their community. Two options for government present themselves: (1) expand the range of EBPs available in the Guidebook, which is difficult until more evidence becomes available about the efficacy of new programs; or (2) grow the knowledge and skill base in program adaptation and evaluation skills to ensure programs are tailored to each community. The latter appears to be a pressing need. Previous research demonstrates that the first step to successful uptake is to explore the reasons for programme adaptations to‐date (e.g. philosophical vs. logistical, time, resources, participant retention), followed by resource planning for targeted education and technical support where needed (Furlong & McGilloway, 2015; Moore et al., 2013).

Learnings from both implementation science and economic geography research suggest a range of recommendations for service providers and government personnel in CfC and similar complex community initiatives. Implementation science research indicates that service providers in thin markets are able to implement EBPs effectively despite challenging conditions where they have made strategic and judicial use of the resources available to them, where they have built local community capacity to co‐deliver programs, and where there is good local leadership (Furlong & McGilloway, 2015; Roufeil & Battye, 2008). Economic geography research suggests service providers can overcome some of the thin market challenges by ‘creating markets’ and efficiencies through networking with nearby services to purchase the programs, personnel, and the associated technical support for implementation (Girth et al., 2012). Yet, the onus for addressing such challenges should not just rest with providers. The policy environment must recognise and respond to the unique service delivery context in thin markets brought about by uneven geographic development. Many studies examining attitudes and implementation facilitators and barriers have found that improving knowledge is not sufficient on its own: resource investment and practical supports are also required for effective and sustained delivery of EBPs (Li et al., 2019). This will help build workforce and community capacity in areas where staff availability is low and turnover is more common. Careful planning and additional support should be considered to assist in the development of regional networks and partnerships for the sharing of skills and expertise and additional intensive support provided where required.

We note some limitations of our study. As there is no central record of the number of services and people who work at CfC, we relied on a snowballing approach for recruitment. Overall, however, we had good participant representation across all states, with adequate sample sizes for both Facilitating Partner and Community Partner organisations. Measures were mostly study‐designed, and all were self‐reported with the possibility of reporting bias: participants self‐rate knowledge higher than objectively assessed knowledge (Snibsøer et al., 2018), and attitudinal self‐ratings can also be unreliable (Lavrakas, 2008). The small sample and wide confidence intervals indicate caution is required when interpreting the findings about remote differences. Nevertheless, the consistency in findings for remote participants, both within this study and against our previous work, tends to support the robustness of the results. Future studies could explore the experiences of community stakeholders and programme recipients (families) to understand their views about the range of programmes on offer.

The notion that place matters when considering the distribution of health services and its impact on health outcomes has been well‐studied. However, many studies tend to focus on local context and ignore broader geographical differences which may also impact on health. Our study shows implementing EBPs in a national place‐based initiative requires careful consideration of geographic factors. The goal of place‐based initiatives is to close the inequality gap between intervention areas and the rest of the population. Yet, well‐intended policies have the potential to entrench or exacerbate inequalities if attention is not also given to the broader scale contextual factors that influence implementation.

## AUTHOR CONTRIBUTIONS

All authors contributed to the study conception and design. The survey was constructed by FB in Qualtrics, with support provided by SH. Quantitative data analysis was performed by FB, with support provided by SH and JN. Qualitative data analysis was performed by FB, with support provided by SC. The draft manuscript was written by FB, and all authors reviewed and edited. All authors read and approved the final manuscript. Supervision for all aspects of this study was provided by SH, SC, NH, and JN.

## FUNDING INFORMATION

Fiona Burgemeister has an Australian Government Research Training Program PhD scholarship. No funding was received for conducting this study.

## CONFLICT OF INTEREST

The authors declare that there is no conflict of interest.

## ETHICAL APPROVAL

Ethical approval for this study was granted by the La Trobe University Human Research Ethics Committee (HEC18198). Study approval was also obtained from the Australian Department of Social Services.

## CONSENT TO PARTICIPATE

Informed consent was obtained from all individual participants included in the study. Informed consent was obtained from all individual participants to publish their data.

## Data Availability

Data available on request due to privacy/ethical restrictions
